# Polypharmacy-Induced Long QT Syndrome in a Patient With Newly Diagnosed Acute Myeloid Leukaemia: A Case Report

**DOI:** 10.7759/cureus.36914

**Published:** 2023-03-30

**Authors:** Zahid Khan

**Affiliations:** 1 Acute Medicine, Mid and South Essex NHS (National Health Services) Foundation Trust, Southend-on-Sea, GBR; 2 Cardiology, Bart’s Heart Centre, London, GBR; 3 Cardiology and General Medicine, Barking, Havering and Redbridge University Hospitals NHS (National Health Services) Trust, London, GBR; 4 Cardiology, Royal Free Hospital, London, GBR

**Keywords:** electrolyte imbalance, chemotherapy response, life-threatening arrhythmia, ventricular tachycardia (vt), severe sepsis, ecg interpretation, rational polypharmacy, long qtc, leukaemia, acute myeloid leukaemia

## Abstract

Long QT syndrome is a type of disease caused by ion channels in the heart not working properly. It is a rare condition that can affect up to one in 2000 people. Many people with this condition do not develop any symptoms; however, this can lead to heart rhythm abnormality, known as torsades de pointes, and can sometimes be fatal. The cause of this condition is often inherited; however, it can also be triggered by certain medications. But the latter tends to affect those who already tend to develop this condition. The medications causing this condition include antiarrhythmics, antibiotics, antihistamines, antiemetics, antidepressants, antipsychotics, and many more. In this case report, we will be discussing a 63-year-old female who developed long QT syndrome as a result of the multiple drug therapy which is associated with long QT syndrome. Our patient was admitted to the hospital with dyspnoea, fatigue, and weight loss and was diagnosed with acute myeloid leukaemia. The patient was commenced on several medications leading to a prolonged QTc interval which resolved after stopping the culprit medications.

## Introduction

The electrical system of the heart controls the heartbeat, and ion channels play a major role in recharging and discharging electrical current. When the ion channels are not working properly, the heart takes longer than usual to recharge between each beat, and this delay can be seen on the electrocardiogram (ECG) as a prolonged QT interval [1].

The QT interval changes with variation in the heart rate: it shortens when the heart rate increases. This makes it difficult to compare the QT intervals measured at different times at different heart rates. In order to compare QT intervals measured at different heart rates, a number of mathematical formulae are used to correct the QT interval at a standard heart of 60 beats per minute. This is known as corrected QT, or in short, the QTc. The normal QTc value for adult men is 350-450ms, and for adult women, it is 360-460ms [2]. If QTc is prolonged than the normal values, this condition is called long QT syndrome, and this can lead to ventricular arrhythmia called torsades de pointes. Moreover, prolonged QTc is an independent risk factor for sudden cardiac death [3,4].

Long QT syndrome can be congenital or acquired. Medication side effects and electrolyte imbalance are the most common causes of acquired long QT syndrome. Low levels of electrolytes such as potassium and magnesium can provoke this condition. As for medications, different classes of medications have been shown to have risk for developing this condition. These include certain types of antibiotics, antiarrhythmics, antiemetics, antipsychotics, antihistamines, chemotherapy drugs, and many more [5]. Herein, we present a case of a 63-year-old lady treated concomitantly with chemotherapy for acute myeloid leukaemia (AML), two antiemetics that are associated with prolongation of the QTc interval, and an antibiotic leading to long QT syndrome believed to be due to the combined effect of these medications.

## Case presentation

A 63-year-old woman, who was previously fit and well with no past medical history, presented to the Accident and Emergency department with fever, shortness of breath on exertion, reduced appetite, increasing fatigue, petechial rash over arm and shin and recent weight loss of about five kilos. The initial blood tests revealed very high WBC with blast cells of >90% on a blood film, raised CRP, low platelets and neutrophil count of 0.6 (Table 1). She was reviewed by a haematologist, and bone marrow biopsy was done, which demonstrated that 87% of total nucleated cells (TNCs) were CD34+ and CD117+ myeloid progenitors. These cells were positive for myeloperoxidase (MPO), and the lineage-associated markers CD7, CD13, and CD33 were positive. These findings were consistent with the diagnosis of AML with minimal differentiation. She was febrile with a temperate of 38.5C and was started on IV tazocin and amikacin for neutropenic fevers of an unclear source. Initial blood cultures, urine cultures and stool examination were all unremarkable. Respiratory viral swabs were negative.

**Table 1:** Lab test result trend during hospital admission

Lab Test	On Admission	Day 11	Day 18	Day 39	Reference Values
Haemoglobin	104	68	75	75	120-150 g/L
White Cell Count	51.7	6.9	0.3	6.4	4-10 x10^9/L
Platelets	16	8	12	69	150-410 x10^9/L
Blast Cells	48.1				0-0.01 x10^9/L
Neutrophil	1.0	0.1	0.0	3.4	2-7 x10^9/L
Nucleated Red Blood Cell	0.7	0.0	0.0	0.2	0-0.2 x10^9/L
Urea	5.6	5.7	1.7	4.1	2.5-7.8 mmol/L
Creatinine	68	68	58	40	45-84 umol/L
Sodium	136	136	136	133	133-146 mmol/L
Potassium	3.8	3.6	3.9	3.7	3.5-5.3 mmol/L
Alanine Aminotransferase	20	13	15	27	0-33 unit/L
Alkaline Phosphatase	106	71	79	400	30-130 unit/L
Bilirubin	5	11	4	14	0-21 umol/L
C-Reactive Protein	44	58	106	42	0-5 mg/L
Calcium	2.28	2.30	2.32	2.26	2.2-2.6 mmol/L
Phosphate	1.16	1.27	0.87	0.29	0.8-1.5 mmol/L
Magnesium	0.8	1.0	0.8	0.8	0.7-1 mmol/L
Thyroid Stimulating Hormone	2.80				0.27-4.2 mU/L
T4 Thyroid Hormone	17.9				10.5-24.5 pmol/L
HBA1C	109				20-41 mmol/mol

**Figure 1:**
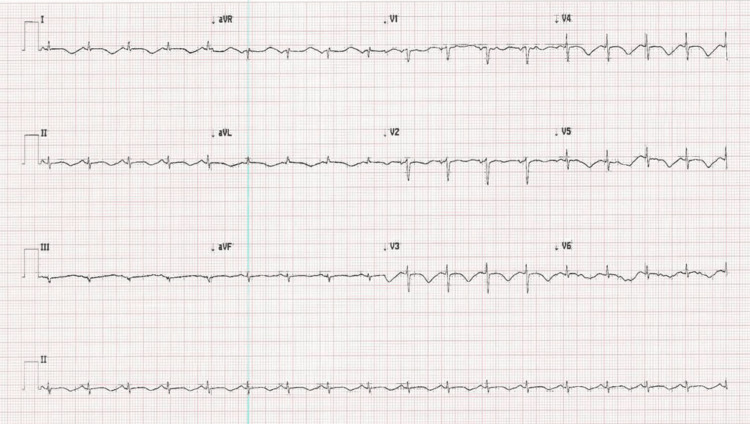
Electrocardiogram showing normal sinus rhythm with T wave inversion in anterolateral leads 1, AVL, V2-V5 and prolonged QT interval

Initial admission ECG showed normal sinus rhythm with PR interval, narrow QRS complexes, inverted/flat inferolateral T waves, and corrected QT interval (QTc) 408ms. Echocardiogram was normal with a left ventricular ejection fraction (LVEF) of 60-65% without any evidence of regional wall motion abnormalities (RWMAs). She was started on hydroxycarbamide and prophylactic aciclovir as per haematology advice. Her HbA1c was noted to be 109, and she had few hyperglycaemic episodes and was reviewed by the diabetes team who started her on insulin, metformin and gliclazide, and her blood sugars were monitored. After gaining consent, the PICC line was inserted, and she was started on chemotherapy with daunorubicin, cytarabine and Mylotarg for AML. On day 3 after the first cycle of chemotherapy, her lab tests results showed Hb 68 (120-150 g/L), platelets 8 (150-410 x 10^9/L), WCC 6.9 (4-10 x 10^9/L), and neutrophils 0.1 (2-7 x 10^9/L) and hydroxycarbamide was stopped. She received packed red blood cells and platelet transfusion to improve her Hb and platelet count. She had further temperature spikes, had few episodes of loose stools, and developed popular rashes on the upper limb. Antiviral therapy was continued, and stool and blood cultures were sent. She became leucopenic and neutropenic and was started on granulocyte colony-stimulating factor and platelets. RBCs were topped up as needed. The patient had vomiting post chemotherapy leading to reduced oral intake and was put on regular ondansetron and domperidone. With her ongoing sickness and diarrhoea, her potassium and phosphate became 2.7 mmol/L and 0.28 mmol/L respectively and were replaced intravenously. Her blood cultures grew Pseudomonas stutzeri, and tazocin was switched to IV meropenem. She continued to spike temperatures, and repeat blood cultures from the PICC line grew Enterococcus faecium which was resistant to vancomycin, so meropenem was switched to daptomycin, the PICC line was removed, and ECHO was suggested with persistent bacteremia. The patient became tachycardic with no chest pain, ECG showed sinus rhythm,105 bpm, deep T wave inversion in lateral leads, PR interval 70ms, narrow QRS and QTc 485ms (Figure 1). She had an echocardiogram that showed moderately impaired LV systolic function with an ejection fraction (EF) of 38% and global hypokinesia from mid to apex, mild mitral regurgitation and normal right ventricular function (Video 1).

 

**Video 1:** Echocardiogram apical 4 chamber view showing impaired left ventricular systolic function

**Figure 2:**
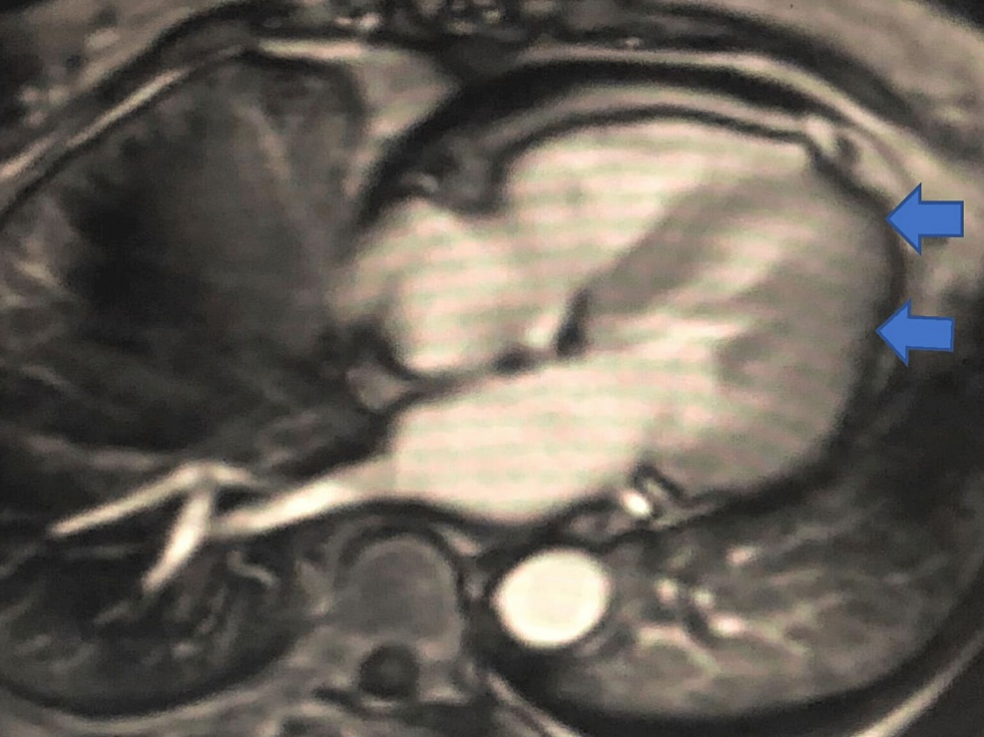
Cardiac magnetic resonance imaging 4 chamber view showing myocardial oedema and fibrosis suggestive of an acute inflammatory process as shown by blue arrows

The patient was reviewed by the cardiology team and was started on bisoprolol, ramipril and furosemide 40 mg twice daily intravenously. The patient had cardiac tomography coronary angiogram and cardiac magnetic resonance imaging (CMR). Cardiac biomarkers showed troponins of 41ng/l and 28ng/L and brain natriuretic peptide (NT-proBNP) 5,736ng/l. ECG showed normal sinus rhythm with a prolonged QTc interval, and both ondansetron and domperidone were stopped. Patient's electrolytes including bone profile were within normal limits and were put on a cardiac monitor. Cardiac MRI revealed acute global LV myocardial oedema with mildly impaired systolic function, global hypokinesia which was more pronounced in the apical segments and mild diffuse fibrosis. CMR did not show any evidence of acute myocardial infarction. Additional findings were that of small pericardial effusion, small bilateral pleural effusions and ascites (Video 2 and Figure 2).

**Video 2:** Cardiac magnetic resonance imaging showing mildly impaired LV systolic function with global hypokinesia, myocardial oedema and trace mitral regurgitation LV: Left ventricular

**Figure 3:**
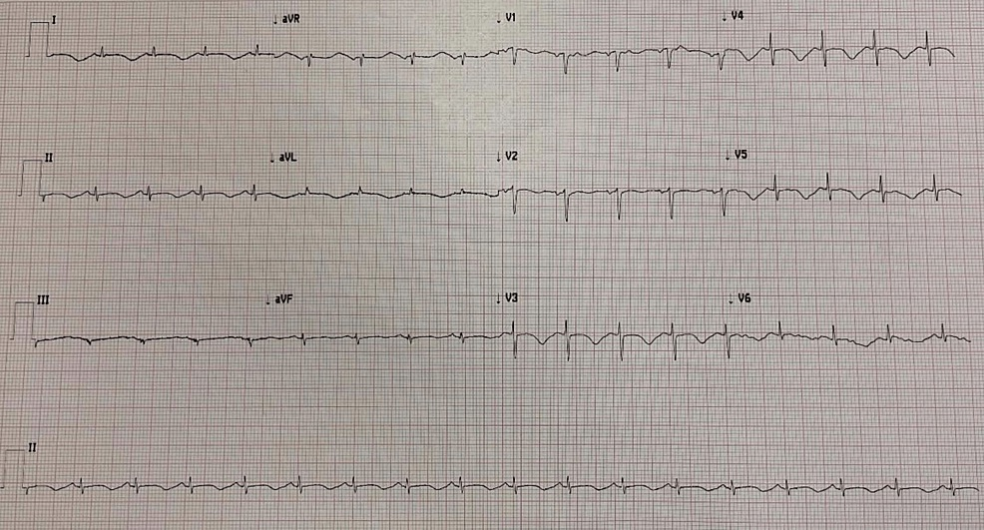
Electrocardiogram showing Deep anterolateral T wave inversion in leads 1, AVL, V2-V5 and prolonged QT interval of 559ms

**Figure 4:**
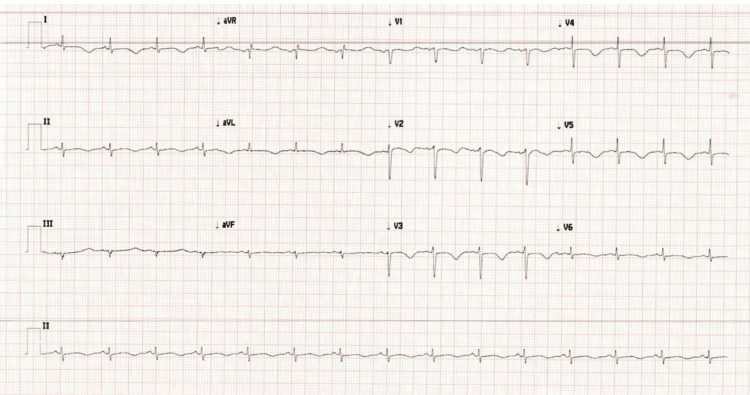
Electrocardiogram showing deep anterolateral T wave inversion in leads 1, AVL, V2-V5 and prolonged QT interval

Subsequently, ECGs showed deep anterolateral T wave inversion, prolongation of QTc at 559ms (Figure 3) and deep anterolateral T wave inversion, and prolongation of QTc at 554ms (Figure 4).

 

**Figure 5:**
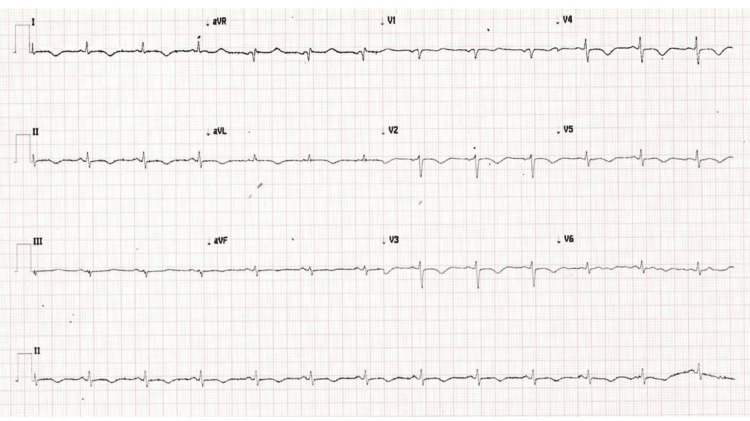
Electrocardiogram showing normal sinus rhythm with anterolateral T wave inversion in leads 1, AVL, V2-V5 and QTc interval of 479ms

After subsequent review of medications, daptomycin was stopped. No obvious runs of VT noted. CT coronary angiogram showed mild three-vessel disease with a calcium score of 181 in LAD and 40 in RCA. It was diagnosed to be likely Takotsubo cardiomyopathy->suspected acute idiosyncratic reaction to chemotherapy, QT prolongation secondary to multidrug therapy. After stopping the offending drugs, the QTc improved with the recent ECG showing sinus rhythm, anterolateral T wave inversion, and QTc of 479ms (Figure 5). The repeat ECHO two months later showed a normal LV cavity with a well-improved LV systolic function of 63% and no obvious RWMA as shown in Video 3.

**Video 3:** Echocardiogram apical 4 chamber view shows normal left ventricular systolic function with no regional wall motion abnormalities

The repeat bone marrow biopsy was done to monitor the outcome following the third cycle of chemotherapy for AML which confirmed the patient to be in remission. The bone marrow biopsy immunophenotyping showed weak to moderate presentation of CD34+, CD117+, and CD13+, and trilineage haematopoiesis did not show any excess of blast cells. The patient's white cell count became normal 6.4 with no blast cells on blood film microscopy. The patient remains under outpatient haematology clinic follow-up and has been in remission since discharge.

## Discussion

The QT interval is the measure of the time between ventricular depolarisation and repolarisation. A prolonged QT interval means delayed myocardial repolarization which can lead to ventricular arrhythmia, i.e., torsades de pointes. The QT interval on the surface ECG represents the summation of action potential of ventricular myocytes which reflects the flow of ion currents through a protein complex-made specialised channels, and malfunction of these channels can result in increased inward or reduced outward current resulting in QT interval prolongation [6]. The QT interval varies with the heart rate, and a number of formulas can be used to calculate the corrected QT interval or the QTc. Nowadays, there are online calculators and telephone applications such as MDCalc which can be used to calculate the QTc conveniently, and we can compare QTc values of the ECGs taken at different times at different heart rates easily. Although the long QT syndrome is said to be rare, the incidence of this condition has been increasing, and many drugs have been shown to be associated with this condition. In fact, it is particularly important to monitor the QTc if a patient is being treated with multiple medications known to cause this condition [6].

This case report emphasizes the importance of monitoring patients for QTc prolongation on polypharmacy such as this patient who developed long QT syndrome as a result of polypharmacy. Antiemetics such as ondansetron and metoclopramide are known risk factors for QTc prolongation. Daptomycin, a novel antibiotic that is very effective in treating Gram-positive bacteria especially methicillin-resistant Staphylococcus aureus and vancomycin-resistant Enterococci, can be associated with this condition [7]. Daunorubicin and cytarabine, a combination chemotherapy that is commonly used in haematological cancers, have been found to have immediate and late cardiotoxic manifestations including cardiomyopathy and QT prolongation [5]. If more than two drugs associated with this condition are used in a patient, the risk will become higher, and this lady was believed to have suffered from the combined effects of points despite these medications. Besides, she also developed cardiomyopathy with reduction in the LVEF which is not certain whether it is due to Takotsubo cardiomyopathy or the side effects of daunorubicin and cytarabine or both. Luckily, the LV function recovered, and she did not develop torsades de pointes and the long QT syndrome.

Patients on medications that can cause QTc prolongation should be monitored on a cardiac monitor and daily ECGs ideally. Daily ECG monitoring is easy to obtain and can be a useful guide to monitor the QT interval in patients on these drugs which allows this clinical condition to be identified early and treated in a timely manner. Any causative medications may need to be stopped, and in case of polypharmacy, the likely culprit and least required medications should be stopped, and the patient should be closely monitored for any dangerous arrhythmia. Various formulas are used to calculate the QT interval such as Bazett, Fridericia, Framingham, Hodges and Van de Water [7]. The most commonly used formula is the Bazett formula; however, it is not reliable in patients with bradycardia or heart rate < 60 bpm as this under-corrects the QTc value and over-corrects it at heart rate > 100 bpm [7].

## Conclusions

In conclusion, prolonged QTc is an uncommon condition with potentially serious complications including arrhythmia. It is important for clinicians to be aware of medications that can lead to this condition, and polypharmacy can cause significant prolongation of the QTc. Our patient developed this condition due to polypharmacy, and the QTc interval normalized when culprit medications were stopped. These patients are at risk of serious arrhythmia and should be on cardiac monitoring.
